# Flame Synthesis of Carbon and Metal-Oxide Nanoparticles: Flame Types, Effects of Combustion Parameters on Properties and Measurement Methods

**DOI:** 10.3390/ma16031192

**Published:** 2023-01-30

**Authors:** Raul Serrano-Bayona, Carson Chu, Peng Liu, William L. Roberts

**Affiliations:** Clean Combustion Research Center, Physical Sciences and Engineering Division, King Abdullah University of Science and Technology (KAUST), Thuwal 23955-6900, Saudi Arabia

**Keywords:** carbon-nanoparticles, metal-oxide nanoparticles, flame synthesis, in-situ measurement methods, ex-situ measurement methods, equivalence ratio

## Abstract

Carbon and metal-oxide nanoparticles (NP) are currently synthesized worldwide for various applications in the solar-energy, optical, pharmaceutical, and biomedical industries, among many others. Gas phase methods comprise flame synthesis and flame spray pyrolysis (FSP), which provide high efficiency, low cost, and the possibility of large-scale applications. The variation of combustion operation parameters exerts significant effects on the properties of the NPs. An analysis of the latest research results relevant to NP flame synthesis can provide new insight into the optimization of these methods and the development of these techniques for a large scale. This review offers insight into the current status of flame synthesis for carbon and metal-oxide NPs—specifically containing analysis and comparison of the most common carbon and metal-oxide NP production techniques. The burner configurations used at the laboratory scale and large scale are also discussed, followed by the assessment of the influence of combustion parameters on the properties of NPs. Finally, the features of the measurement techniques applied for determining NP properties were described.

## 1. Introduction

Nanoparticles (NP) are defined as those particles with a size lower than 100 nm [1], which are featured with a spherical shape and a composition of natural or artificial polymers [2]. Among the entire group of elements that can compose a nanoparticle, carbon and metal-oxide (TiO_2_, SiO_2_, Fe_x_O_y_, Al_x_O_y_, etc.) molecules are most often found and studied, following their advantages in potential applications--their spherical shape and high surface area-to-volume ratio [2]. The following is a brief discussion of the abundance and applications of different NP, as well as the methods that yield them.

Carbon nanoparticles (CNP) are identified with unusual mechanical, electrical, and chemical properties [3,4,5]. The most notable of these nanostructures are carbon nanotubes (CNT) and carbon nano-onions (CNO) [4]. CNTs provide an alternative to use for composite materials reinforcement, fuel cells, Li-ion batteries, and hydrogen storage [3]. Because of their high specific surface area and electrical conductivity [6], CNOs have been considered for use in solar and fuel cells, solid lubricants, and catalytic materials. One concern regarding the synthesis of CNPs is their tendency to agglomerate [5]. Titanium-based nanomaterials have become essential in applications related to energy and water [7], because of their use as components for photovoltaic, photocatalytic, and electrochemical processes [7,8]. Synthesized TiO_2_ materials have four different phases: anatase, rutile, srilankite (TiO_2_-II) and brookite [8,9]. The most frequently used TiO_2_ NP is the Degussa P25, which is composed of 20% rutile and 80% anatase phases [9]. The anatase phase is characterized by a low rate of charge recombination [9,10]; the rutile phase has strong thermodynamic stability [9], and it is one component of the white pigments [10]. The srilankite phase has been identified with an open crystal structure, metastable at high pressures [8]. Iron-based NPs offer functional magnetic properties and high chemical reactivity [11,12,13], including the possibility for use in magnetic resonance imaging, magnetorheological fluids, and microfluiding systems [12]. Their chemical reactivity also enables their use in lithium-ion batteries, and catalysis [12,13]. These NPs are commonly found in magnetite (Fe_3_O_4_), maghemite (γ-Fe_2_O_3_), and hematite phases (α-Fe_2_O_3_) [11,12,13]. The wustite phase (FeO) [12,13] has also been identified in these synthesized materials.

Silica-based NPs are one of the most synthesized materials [14], widely used in the pharmaceutical and medical industries [15]. However, this particular use has been questioned recently since there is new evidence regarding the toxicity of this material [14]. Ceria-based nanoparticles are useful in biomedicine since they present properties similar to conventional antioxidant enzymes [16].

Tungsten-oxide nanoparticles are promising for the fabrication of photoelectrodes because they present high electron mobility and a bandgap satisfactory for this purpose [17]. Aluminum-based nanoparticles (Al_2_O_3_) have also been studied, and relevant features discovered, such as their crystal structure, phase transformations, and useful properties, such as thermodynamic stability [18]. These features are excellent for application in optics and electronics, in catalysis, and as adsorbent, coating, and abrasive materials [18]. Synthesized Al_2_O_3_, with thermodynamic stability, is known as the corundum (α-Al_2_O_3_) [18]; however, metastable transition phases have been found in the production of these NPs, some of them are named γ-, η-, ρ-, θ-, δ-, χ-, and κ-Al_2_O_3_ [18].

Currently, the most synthesized NPs in use (also recognized as engineered nanomaterials (ENM) referring to a large scale [19]), are the carbon black, with an estimated production of 9.6 [millions of metric tons/year], followed by silica-based NPs whose production was ranked at 1.5 millions of metric tons/year by 2010 [19]. By the same year, the United States synthesized nearly 50% of the world ENMs. The highest regional use of the ENMs in the automotive, electronics and optics, sensors and tires industries occurred in Asia, with a consumption of nearly 38% of the total use in each of the aforementioned industries [19]. In the cosmetics industry, the highest use of ENMs was registered in Europe, with 37% of the entire consumption of synthesized NPs [19]. In 2016, the global market of ENMs was valued as USD 7.3 billion [20], and the exponential growth of this value per year was observed, in both pessimistic and optimistic scenarios [21].

Several processes and technologies are used for NP synthesis; these methods can be sorted into two main groups, top-down methods and bottom-up methods [1,22,23]. Top-down techniques are based on the conversion of bulk material into smaller particles in the nanometer scale [22]. These methods are considered to be physical, and this category includes mechanical milling, laser ablation, arc-discharge, hydrothermal, sputtering, chemical etching, and electro-explosion techniques. This approach also includes evaporation and condensation processes in macro-scale [1,22,23]. The bottom-up category involves those methods that begin formation of NPs from atoms and molecules [22]. This approach is comprised of chemical methods in the solid state (physical vapor deposition, chemical vapor deposition), in the liquid state (sol–gel methods, chemical reduction, solvothermal method), in the gas-phase (flame-spray pyrolysis, laser ablation, plasma or flame synthesis), biological synthesis (bacteria, yeasts, fungi, algae, plants) and other techniques (precipitation, ultra-sound, microwave, etc. [22]). For carbon nanomaterials, the most conventional method is chemical vapor deposition (CVD) [3,4,24,25,26], although there are other methods such as arc discharge and laser ablation [3,4,25,26]. Metal-oxide nanoparticles, including TiO_2_, SiO_2_, Fe_x_O_y_, and Al_x_O_y_, can be produced using the Stöber method [14] and precipitation [27]. Plasma or wall-heated reactors are also used as heater sources [8,28].

CVD processes are well known for their ability to produce large scale CNTs, highlighting the variation of floating catalysts CVD (FCCVD), and presented as an alternative to synthesis, with control of nanoparticle production and process continuity and scalability [26]. CVD’s are commonly used in the production of iron-oxide nanoparticles [11]. Shortcomings of the chemical methods include the difficulty in disposing of the residuals, and the impurity in the materials obtained, observed for Al_2_O_3_ production through precipitation (i.e., the Bayer process) [18]. Consequently, the particles produced by these techniques require high temperature calcination treatment to increase their purity [27], and improve the crystallinity of the NPs obtained [17]. The effect of sintering causes these high temperature treatments to sacrifice their surface area [27]. Moreover, the secondary process used for nanoparticle production increases the cost of synthesis.

The gas-phase methods include flame synthesis and flame spray pyrolysis [13,14]. The use of these techniques for production of NPs allows control of operation parameters in order to obtain materials with specific properties [8,10,12,13]. Laminar flames are widely used in laboratory scale [8,10], and the configuration may be premixed or diffusive [8]. However, large-scale burners are used in the turbulent regime for mass particle production [10]. As noted, combustion parameters influence the growth of the nanomaterials; this growth differs significantly from laminar to turbulent regimes in flames [10].

Flames based on hydrocarbon fuels represent a continuous carbon source, which—in addition to the autothermal conditions in the flame—provides a more highly efficient process compared with CVD in the production of CNPs [3,24]. This process can also be performed with less expense, since low energy consumption and simpler devices are used [4]. Currently, the flame synthesis method is the main technique used worldwide for fabricating titanium-based nanoparticles on a large scale [7].

In certain metal-oxides and carbon NPs, combustion operating parameters significantly influence their properties (e.g., structural phase) [11,12,18], as shown in alumina NPs [18], and iron-oxide nanoparticles [13], with special consideration for the hematite phase [12] and the CNTs, where an analysis of the effects on their features from the sampling substrate [3], and the fuel structure [4,25] were conducted. Turbulent flames also improved the production of NPs at a large scale. A performance analysis of the flame synthesis using this configuration in a smaller scale (lab-scale) proved remarkable [10].

This review offers a comparison of varieties of flame configurations on synthesis of carbon- and metal-based nanoparticles (NPs), followed by a description of the effect of operation parameters on the features of the particles produced, offering information regarding the properties of these materials and the operation conditions of the process used to achieve them. This work also analyses the in situ and ex situ measurement techniques for the materials obtained.

## 2. Flame Configuration

A flame provides thermal energy to evaporate particle precursors and initiate chemical reactions that allow particles to grow. Various burners have been used to accommodate engineering needs (e.g., production of particles of different properties, ease of modeling, ease of diagnostics, etc.). Usage, advantages, and disadvantages of different burners are examined as follows. Table 1 provides summarized information on the flame configurations used in studies of nanoparticle flame synthesis.

### 2.1. Premixed Flames

**Figure 1:**
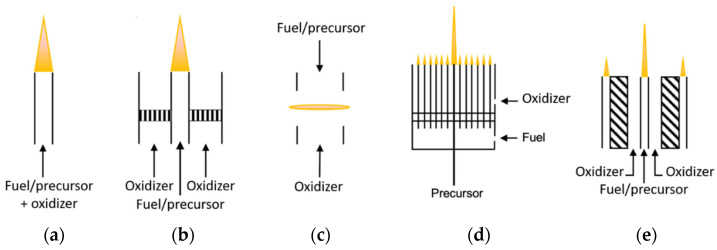
Flame configurations commonly used for flame synthesis process: (**a**) premixed flame, (**b**) normal diffusion flame, (**c**) counterflow diffusion flame, (**d**) multiple diffusion flames, (**e**) flame spray pyrolysis.

In premixed Bunsen burner flames, gaseous fuel or a precursor mixes with an oxidizer and fuel before burning (as shown in Figure 1a). Radial temperature profiles are uniform in premixed laminar flames. Varying the fuel stoichiometry in premixed flames can control the production of species in the post-flame region [29,47]. Premixed flames are subject to the risk of flashback, which occurs when the flame enters and propagates through the burner [48]. Flame arrestors and safety valves should be installed in the fuel lines to avoid flashback; pyrophoric fuels should be avoided to minimize the risk of flashback [30]. If pyrophoric fuels are studied, pressure should be reduced to minimize the formation and accumulation of particles within the burner tube [30]. However, in practical devices, mixers may be required for different degrees of premixing—increasing the complexity of the devices [48].

### 2.2. Non-Premixed Diffusion Flames

In non-premixed flames, the fuel/precursor and the oxidizer are fed separately into the reactor; this configuration eliminates the hazard of flashback in premixed flames and allows a wider range and operating conditions. However, unlike premixed flames, non-premixed flames possess rather complex geometries. Decoupling the flame properties—including temperature, equivalence ratio, products, and residence time—is challenging.

Arranging the distribution of the fuel/oxidizer streams enables different diffusion flames (namely normal diffusion flames, inverse diffusion flames, and counterflow flames).

#### 2.2.1. Normal Diffusion Flames

A co-annular burner, consisting of an inner fuel tube and a concentric outer oxidizer tube (Figure 1b), can produce a normal diffusion flame. As the fuel flows along the axis, it diffuses radially outward, while the oxidizer diffuses radially inward [48]. The fuel and oxidizer react stoichiometrically in the narrow regions, forming flame zones. The radial temperature profile is typically annular-shaped, with high temperatures on the sides and low temperatures in the center. The fuel flow rate primarily controls the flame length [49,50]. Soot—an undesirable product in flame synthesis—forms on the fuel side of the reaction zone of hydrocarbon flames due to high temperatures; it is oxidized and consumed as it travels downstream into an oxidation region. Soot can grow along the metal particle, forming a carbon layer; however, depending on the flame conditions, soot may break through the flame, forming a sooting or smoking flame. The flames, in general, have higher flame temperatures and produce nanoparticles with larger sizes [31,32]. For the same reason, this flame configuration is unsuitable for low melting temperature substrates, e.g., silicon substrates with anode aluminum oxide nanotemplates [33].

#### 2.2.2. Inverse Diffusion Flames

Inverse diffusion flames and normal diffusion flames can share the same burner geometry. Unlike the latter, the inner tube supplies the oxidizer of inverse diffusion flames into a fuel reservoir. In inverse diffusion flames, polycyclic aromatic hydrocarbon (PAH) and soot form outside the reaction zone [51]. The flames contain low soot concentration [34], which is beneficial for the production of carbonaceous particles (e.g., carbon nanotubes (CNT)), as the competition from soot formation is minimal. Unrau et al. [35] reported that using oxy-fuel inverse diffusion flames minimizes the contamination of amorphous carbon impurity. Carbonaceous particles form on the annular side of flames, avoiding the particle oxidation and thermal defects induced by the flame high-temperature region [36,47]. This formation also facilitates the external collection of the particles [35]. Due to lower flame temperatures, inverse diffusion flames may produce smaller nanoparticles than normal diffusion flames [31,32].

#### 2.2.3. Counterflow Diffusion Flames

Counterflow diffusion flames employ two opposing jets of fuel and oxidizer (Figure 1c). A stagnation plane formed when the axial velocity equals zero creates two distinctive regions, fuel-rich and fuel-lean, on the fuel and the oxidizer sides. The flame forms on the oxidizer side of the plane. The flames are used mainly in fundamental research because they approximate a one-dimensional character, making measurements and calculations much more practical [48,52]. The one-dimensionality of the flames also gives flexibility in adjusting the spatial gradient of temperature and reactant concentration [38], enabling a fundamental study of the effects of temperature, catalytic substrate positioning, and gas-phase composition on synthesis processes [47]. In addition, the flames allow minimum flame-substrate interaction, facilitating specific particle production processes. For example, Li et al. used silicon substrates with anode aluminum oxide nanotemplates, sensitive to temperature change, in a counterflow flame to produce well-aligned, size-controllable CNTs [33]. The synthesis zone of CNTs is on the fuel side near the flame edge due to the closer supply of carbon. Nanoparticle synthesis in counterflow flames is also affected by the strain rate, which affects the amount of carbon supply and residence time. A counterflow flame with a high strain rate would have more carbon sources but a shorter residence time. However, the availability of carbon sources explains the effect of strain rate on the growth of carbonaceous particles, as opposed to residence time [37]. Thus, although they have a shorter residence time, flames with higher strain rates produce more carbonaceous particles.

#### 2.2.4. Multiple Diffusion Flames

A multi-element diffusion flame burner (MEDB), commonly known as a Hencken Burner, generates multiple-diffusion flames [30,53]. The burner comprises an array of hypodermic needles within a honeycomb [30] (Figure 1d). The fuel flows though the needles, and the oxidizer flows through the surrounding open cells, generating multiple small, tightly spaced diffusion flames. The flames share the advantages of both premixed and non-premixed flames, which are beneficial for the production of nanomaterials. First, uniform temperature and species profiles can be obtained downstream of the flames [39]. Second, as fuel and oxidizer are initially separated, flashback is avoided, as well as unwanted reactions before the flame [39], enabling a wider range of operation and allowing the use of pyrophoric fuels.

### 2.3. Flame Spray Pyrolysis

Flame spray pyrolysis (FSP), mainly used for synthesizing metal oxides [54], refers to a combustion process in which a precursor is in liquid form (a mixture of metal precursor, e.g., nitrates, and organic solvent, e.g., ethanol) and contributes more than 50% of the total energy of combustion [43]. The liquid is atomized and conveyed by the central nozzle, surrounded by a coflowing oxidizer. An external flame is required to provide a heat source to evaporate and ignite the liquid precursor [44,46] (Figure 1e). Because the liquid precursor is highly exothermic, the resultant flame is self-sustaining [54] and has a high temperature [44]. Induced by the entrainment of the surrounding oxidizer, FSP is also characterized by short residence time and steep temperature gradients [45], with rapid cooling of several hundred K ${\mathrm{cm}}^{-1}$ [54]. These factors yield highly homogeneous and crystalline nanomaterials [46].

The composition of liquid precursors can control the morphology of nanoparticles. Karthikeyan et al. [42] showed that nitrate precursors produced crystalline and dense nanoparticles. The use of organometallic precursors can also improve the homogeneity and provide smaller grain size distribution [42]. The oxidizer flow rate is also influential in the morphology of nanoparticles. For small, non-agglomerated particles, the oxidizer flow rate should be high for sufficient mixing, whereas, for larger particles, a reduced oxidizer must be used for a flame with a longer length and residence time [40,41].

## 3. Effect of Operation Conditions in Combustion

Table 2 summarizes the analyzed combustion parameters in recent studies involving the production of NPs through flame synthesis, providing information on the flame configuration, the type of synthesized NP, and the affected particle properties in each study.

### 3.1. Equivalence Ratio in the Flame

The equivalence ratio (φ) is defined as the ratio of the flow rates of fuel and oxidizer applied in the real process, divided by the same ratio at stoichiometric conditions [63,64]. This ratio helps to determine whether the fuel and the oxidizer supplied for the combustion process are under conditions of lean (excess of oxidizer, φ < 1), rich mixtures (excess of fuel, φ > 1), or under stoichiometric conditions (φ = 1). This ratio varies with the change in the flow rate of the oxygen and fuel streams [18]. 

For the determination of combustion performance, this factor is the most relevant in the many processes and devices that use it [48]. In combustion processes, the reactor releases heat, which can be expressed as the enthalpy of reaction (with the same magnitude as the enthalpy of combustion), which varies with the amount of fuel and oxidizer provided in the reaction—and consequently is a function of φ [48]. Furthermore, with the correct assumptions and applying the concept of the first law of thermodynamics, the enthalpy of the reactants at the initial state is equal to that of the products at the final state, featured with the adiabatic flame temperature [48]. Henceforth, the equivalence ratio exerts a remarkable effect over both the enthalpy of combustion and the flame temperature, as evidenced in previous studies on flame synthesis of nanoparticles [10,18,26].

It is possible to control the yield of produced nanoparticles with the variation of φ by considering a negative correlation since oxygen availability dominates the main chemical reaction [15]. The higher amount of oxygen in the synthesis increases the efficiency of the oxidation of precursors and solvents [13].

**Figure 2:**
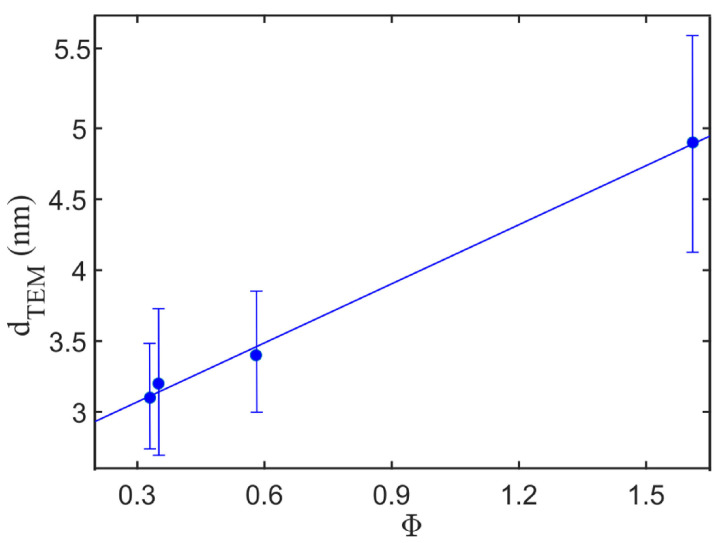
Primary particle sizes of iron-oxide NPs, measured from the micrographs obtained through the application of the transmission electron microscopy (TEM), as a function of the equivalence ratio [12].

Particle size can be controlled by changing the values of φ with a positive correlation in flame synthesis [15,18]. Figure 2 exhibits this correlation for iron-oxide NPs, measured in the study of Sorvali et al. [12]. Furthermore, at values of φ larger than one, the intermediate species in the formation pathway of NPs are identified as oxygen deficient. Consequently, the particles at the end of the synthesis have a high concentration of oxygen vacancies (OVs), resulting in amorphous structures [55].

**Figure 3:**
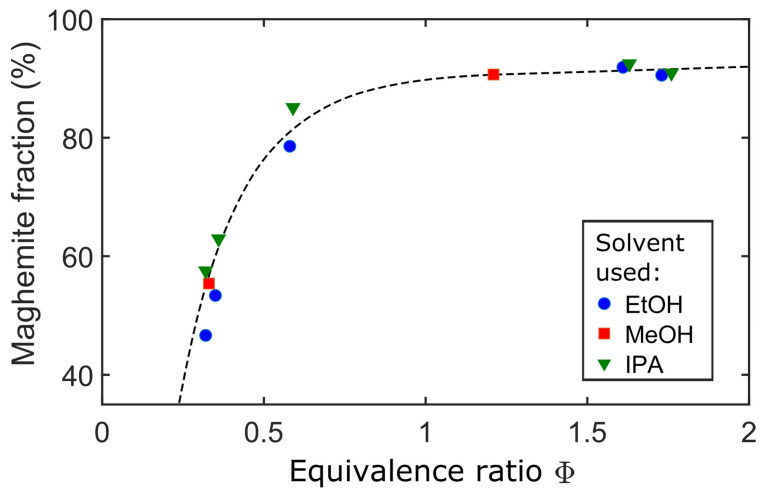
Mass content of the maghemite phase in flame-synthesized iron-oxide NPs as a function of φ, using different alcohols for the precursor solution [12].

The oxygen availability is also a relevant feature in the phase composition of particles, demonstrated in iron-oxide NPs by the reduction of magnetite and the rise in hematite content in the materials produced by the decrease in φ [13]. Sorvali et al. [12] demonstrated the strong effect of φ on the maghemite content in synthesized iron-oxide NPs, regardless of the type of alcohol used as a solvent for the precursor solution (Figure 3).

### 3.2. Effect of Stoichiometric Mixture Fraction

The stoichiometric mixture fraction, Zst, represents the mass fractions of products from the combustion reaction, based on the fuel stream, at the location of complete theoretical combustion. The value of Zst increases at higher oxygen concentrations in the oxidizer stream of a diffusion flame configuration [56]. 

The flame temperature correlates positively with Zst; consequently, this parameter exerts influence over the flame structure, which associates the distribution of chemical species present in the combustion process with the temperature field in the flame. Therefore, Zst also rules the flame shape [56]. Another feature influenced by this fraction in a diffusion flame is the local atomic carbon-to-oxygen ratio (C/O), which indicates the potential of oxide formation in the combustion process. Metal oxides with more oxygen atoms are formed at smaller C/O ratios, while flames with larger C/O ratios synthesize more reduced oxides [56]. 

The relations described above explain the effects on the synthesis of nanoparticles. At first, higher values of Zst in the flame partially suppress the soot formation in hydrocarbon flames, which is convenient for synthesizing metal-oxide NPs [56]. Moreover, the Zst may influence the structural phase composition in NPs, as shown by the formation of iron-oxide NPs, where particles with higher magnetite content were obtained when Zst increased [56].

### 3.3. Effect of Flame Temperature

The flame temperature can be affected by the equivalence ratio (φ) in premixed flames and the stoichiometric mixture fraction (Zst) in diffusion flames, as discussed in Section 2.1 and Section 2.2, respectively. Moreover, the addition of diluent gases (e.g., Ar, N_2_, CO_2_) in a burner and the variation in their flow rates enables the control of the temperature field in the flame [65]. In normal diffusion flames, the reduction of flame temperature is demonstrated by the increase in the velocity of the oxidizer stream, raising the flow rate of cold gases [61], while the increase in the flow rate of the fuel stream supplies higher enthalpies, producing higher flame temperatures [61]. Moreover, the addition of metal-oxide NP precursors also influences this parameter [60] since these are involved in the combustion kinetics, acting as catalysts with OH and H radicals, demonstrated in the synthesis of iron-oxide NPs [60]. 

**Figure 4:**
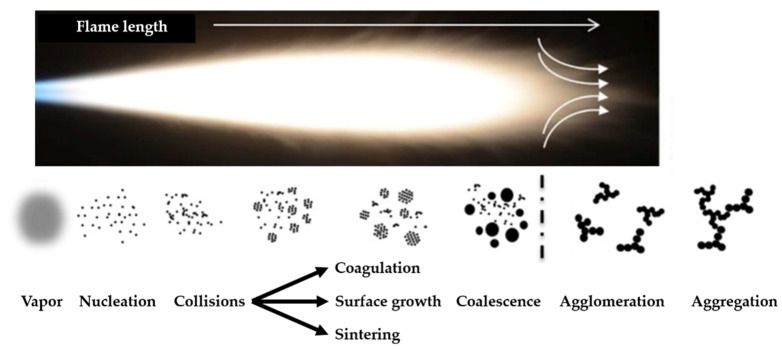
A common pathway of particle formation in flame synthesis processes, as the molecules pass through the flame flow field [66]. Reproduced with permission from Roller, J. et al., Journal of Power Sources; published by Elsevier, 2014.

An increase in the flame temperature reduces the particle size of CNPs [57]. The particle formation of CNPs in flames explains this behavior. Figure 4 exhibits a common particle formation pathway of the flame synthesis process [66], suitable for discussing the evolution of flame-made nanoparticles passing through the flame. The nucleation mode takes place as a single mode from the base of the burner until a particular value of HAB, where the size growth mode (particle coagulation and aggregation) starts and converts the formation process as bimodal [57,58]. As the temperature increases, the HAB limit for the nucleation mode moves towards higher HABs, suppressing the size growth modes of CNPs [57,58]. Moreover, CNPs precursors, such as the PAHs, decompose at high flame temperatures [57,58]. The temperature effect is different for the metal-oxide NPs since this size correlates positively with the flame temperature [56,61]. The positive correlation of the crystal growth rates, particle sintering, and particle collision with the flame temperature justify this behavior [56,61]. Low temperatures increase the time required for sintering and decrease particle collision, leading to lower particle growth rates [61]. Enhanced sintering provides high yields of larger spherical particles [61]. The sintering process is different among the produced metal-based NPs, based on the temperature required for the total coalescence of particles in flame synthesis [61]. As a comparison, titanium-based NPs require lower temperatures for this coalescence, hence obtaining larger particle sizes than silica NPs [61].

High-temperature zones in the flame are responsible for the reduction in the mass concentration of flame-made NPs, [57,60] due to the oxidation of the formed particles at the initial stage [60] and the suppression of the surface growth mechanisms at high temperatures [57]. However, particle-particle coagulation dominates the number density of soot particles. This coagulation process faces thermodynamic reversibility at high temperatures. Therefore, the number density of soot particles is nearly constant. Instead, particle residence time mainly affects this number density [57].

In terms of the morphology of particles, higher temperatures increase the size of the undisturbed lattices [58] and decrease the atomic hydrogen content [57,58]. This last content (also expressed as the H/C ratio in the produced CNPs) leads to a lower susceptibility to oxidation [57]. These facts demonstrate fewer disordered structures in the CNPs synthesized at higher temperatures, which are noticed for particles with constant size [58]. Since the temperature variation dominates the sintering process, temperature also governs the surface area of the obtained particles, demonstrated by the reduced area from decreased temperature [18]. Because this is linked with the combustion enthalpy, it is crucial for forming homogeneous particles [26,62]. Lower values of temperature and enthalpy in the flame enhance a lower degree of sinter neck formation, producing more fused nanoparticles [14,18].

The stability of the structural phases of nanoparticles varies with the temperature in the flame synthesis process. Therefore, the phase transformations occur at high flame temperature increase [15,18]. Moreover, this stability is also ruled by the particle size in both phases, evident in titanium-oxide NPs since the anatase phase is more stable at smaller sizes [10,59], and rutile phase stability improves at larger sizes [59]. Temperature also plays a relevant role in the alumina-based NPs, with the increase of θ-alumina and the reduction of η-alumina phases caused by a temperature increase in the flame [18]. These two phases are related because at higher temperatures, the γ and η-alumina phases transformed into δ-alumina, which under long residence times consequently turned into θ-alumina [18].In iron-based NPs, Fe(III) is almost the unique component at relatively low flame temperatures [56]. This decrease as the temperature rise, leading to a higher content of Fe(II) in the produced material. The increase in the Fe (II) produces higher contents of the magnetite phase [56]. 

### 3.4. Effect of Fuel Type and Precursors

In a combustion process, the heat released also depends on the type of fuel supplied to the burner. Each fuel has a unique heating value, also known as the enthalpy of combustion [48]. Precursors must be added to the reaction to synthesize nanoparticles because they contain the metal components that are oxidized to form these materials--excluding the carbon nanostructures. These precursors can be added to the fuel stream as a solution, considering the main precursor as the solute (e.g., TMS for SiO_2_ and TTIP for TiO_2_) and the use of alcohols as solvents [18]. This solution plays a relevant role in the enthalpy of the reaction [18]. However, this effect is not present in the Hencken burner configuration since the contribution of the combustion heat from the solvents to the flame enthalpy is low [67].

A high solvent boiling point makes possible the formation of homogeneous particles [12,62]. The increase in the precursor feed rate also raises the total number concentration of flame-synthesized particles [13,18] and shifts their distribution to smaller sizes [18]. Low precursor flow rates produce fuel-lean combustion, leading to incomplete oxidation of the components caused by low temperatures [13]. 

Since the most common fuels applied in combustion devices are hydrocarbons (CxHy), these are used as precursors in synthesizing CNPs. The higher number of carbon atoms in the fuel, and the presence of C=C double bonds in the hydrocarbon molecule, accelerate soot formation [25], leading to a much higher amount of soot, as observed in the flame synthesis process using ethylene and compared with the yield obtained in methane flames [25].

### 3.5. Effect of Residence Time

Residence time is defined as the time that particles and molecules last in the reaction zone (bounded by the flame), and can be quantified as the mass of the entire mixture, which is the density of the mixture multiplied by the volume of the reaction zone, and divided by the overall mass flow rate passing through the reactor [48]. The mass flow rates of gases supplied to the burner determine this parameter, also modifying the gas velocity in the process. The residence time increases with the reduction in the carrier gas or oxidizer gas flow rates in the combustion process [13,18]. In the case of a premixed flame, the equivalence ratio plays a relevant role at this time, since a linear influence from φ over the flame height has been shown [12,18] which is more remarkable at low values of carrier gas flow velocity [26]. Additionally, at the same values of φ, the increase in the gas velocity increases the flame height and the particle residence time in the high-temperature zone [12]. The effect from the type of fuel has also been noted, since a higher amount of carbon atoms in the fuel molecules produces a higher residence time in the particles in the flame [10].

The particle size of flame-synthesized nanoparticles is inversely dependent on the velocity of the premixture issuing from the burner and, therefore, the residence time in the synthesis flow field [8,12]. This time is one of the factors governing the sintering process, thus affecting the surface area of the synthesized materials, described as an inverse correlation [10,18]. Furthermore, this time influences the yield of particles with homogeneous structures --with a positive correlation--as evidenced in the production of CNTs through the *I_G_*/*I_D_* ratio [26]. However, it is suggested that to effectively carry out precursor decomposition and solvent combustion, droplet size, and oxidant availability are more relevant than increased residence time [13].

Nevertheless, at short residence times in the flame high-temperature region (which can be caused by the use of low precursor flow rates), the phase transition in nanoparticles is a kinetically limited transformation process (as demonstrated in alumina NPs [18]) since the residence time to carry out a complete conversion of droplets is minimal [13]. This effect is also notable for titanium-based NPs. As the residence time decreases, the rutile yield shrinks, and the srilankite content grows [8]. The increase in anatase content can be explained by a longer residence time in the high-temperature region at a low precursor flow rate [10]; if this time increases, the anatase content mutates to the rutile phase [10].

### 3.6. Effect of Substrate Materials

Substrates are components in the flame synthesis setup where the nanoparticles are deposited after they are formed [25]. These substrates can also carry catalysts to improve the reaction performance [26]. The choice of material for the substrate may affect the synthesis process in terms of purity and crystallinity [3]. The effect of the substrate material arises from three factors linked to their properties [3]. One is the melting point because materials with a lower melting point enable an active state in the substrate, allowing the dissolution of the NPs formed in the substrate and improving their nucleation and growth [3]. Potential oxidation of the elements that compose the substrate is another relevant factor since an oxidizer is essential in the flame synthesis process, and this oxidation inhibits nucleation and growth of NPs [3]. Finally, the substrates with an open spatial configuration permit oxidation of the synthesized NPs, compared with those with a closed space—such as the foam; this is undesirable for some types of nanoparticles (e.g., CNPs [3]). The last factor also affects the morphology of the material obtained, since a closed space can cause disordered structures [3]. This spatial configuration also determines the yield of the synthesis, considering the deposition of NPs as a coating over the substrates. Evidence of deactivation of the catalysts was present in this substrate after its complete encapsulation by the formed NPs [11,25].

### 3.7. Effect of the Flame Configuration

**Figure 5:**
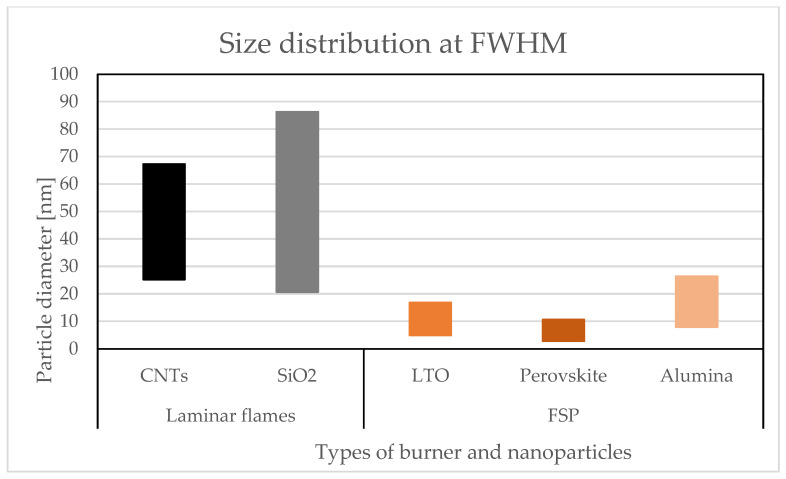
Comparison of particle size distribution obtained for carbon nanotubes (CNTs) [25], silica nanoparticles (SiO_2_) [15], Li_4_Ti_5_O_12_ (LTO) [62], perovskites with added iron particles [27], and alumina NPs [18].

This configuration exerts a notable influence over particle size distribution in the obtained particles, regardless of the operation parameters in the combustion process, and observed in Figure 5, where the interval of the size distribution of NPs obtained through laminar flames is compared with that obtained using the flame spray pyrolysis process. These intervals were obtained from the measured size distributions, using TEM [15,18,25,27,62] extracted through the full-width at half-maximum method (FWHM), which is applied to Gaussian peaks using several measurement techniques [6,13]. Note the larger flame sizes (obtained in laminar (premixed and diffusion)) than those measured in particles synthesized through flame spray pyrolysis (FSP). The gas-to-particle formation route governs the synthesis process in the FSP reactors, which leads to small particle sizes [18]. However, because the FSP configuration operates with short residence times [45], it enables the production of NPs with smaller particles than those obtained through laminar flames, confirming the effect of residence time over particle size (see Section 3.5).

## 4. Measurement Techniques

The following section introduces the most common methods used to characterize flame-synthesized nanoparticles. The section has two subsections: ex situ and in situ measurement techniques. Ex-situ measurements require sample extraction from the reaction or post-flame regions, whereas in-situ measurement techniques are carried out within the flame flow field. 

### 4.1. Particle Collection and Preparation of Samples

Ex-situ measurement techniques generally use two particle collection methods [13]. One is the particle sampling from the reactor filter, and the other can be performed with a steel mesh placed downstream of the reactor [13]. Both forms of sampling are known as non-intrusive, and may become intrusive when the mesh is placed inside the flame zone. Studies focused on the synthesis of CNPs collected the samples mostly using the mesh method [3,4], while the metal-oxides NPs produced at lab scale and assisted by flame were taken more often by the filter technique [10,16,18]. Carvajal et al. assessed both techniques for collecting iron-oxide NPs [13], showing agreement in sizes for both samples (although there was a notable improvement in the dispersion of the particles for the mesh methodology, making it possible to measure individual particle diameters). This improvement was made possible by sampling with the mesh for 1 min, thus obtaining a sub-monolayer of dispersed particles [13]. 

Partial particle fusion may produce the agglomeration of particles during the flame deposition process [68]. For the measurement of individual particles, the materials obtained can be immersed in an ethanol solution [3,12,14,24,25], or diluted in Milli-Q water [68], then sonicated and placed on a grid of holey carbon [24] or copper [3,14,25,68]; finally, these samples are dried. Homogenization or ball milling can improve the dispersion of particles [68]. The strongly negative surface charge of the CNP enhances the dispersion stability via electrostatic repulsion [68]. 

The samples can also be prepared directly from the flame reactor via thermophoresis [62]. This technique is the principle of a cold wall located in the flow field of a gas that carries the synthesized particles, surrounded by a temperature gradient [69]. Here, the flame temperature generates the gradient [69]. This principle states that the exposure time of the probe should be short enough to hold the low temperature on the surface for the produced particles and long enough to gain a significant amount of material from the flame [69]. The samples collected through this method have been analyzed using imaging techniques [69,70,71]. 

Moreover, the synthesized CNPs can be coated with a gold or platinum layer with a thickness of less than 10 nm [24,68], which enhances the appearance of the amorphous carbon layer (ACL) and makes the layer between the CNT forest and the ACL much more distinct [24], also producing a high-resolution image and reduction of surface charging [68]. This coating is applied for the performance of the field emission scanning electron microscopy (FESEM) technique.

### 4.2. Ex-Situ Measurement Techniques

#### 4.2.1. Imaging Techniques—Electron Microscopy

The following imaging techniques are based on the interaction of electrons with the sample. To better comprehend these methods, a brief explanation of the mechanisms that govern these interactions follows. Electrons that target the sample and follow an individual trajectory over it are the primary electrons [72]. Due to the high kinetic energy of the incident primary electrons, secondary electrons (SE) come off the surface of the sample [72]. The primary electrons scattered in angles between 90 and 180 degrees are known as backscattered electrons (BSE) [72]. When a primary electron knocks out an electron of an atom, an electron from a higher energy state subsequently fills the vacancy, generating an X-ray [72,73].

Primary electrons interacting with the matter may have experienced elastic or inelastic scattering; the former occurs when there is no energy loss in the incident primary electrons. In addition—although the direction of scattered electrons may change—their wavelength remains unchanged [72]. In contrast, the latter occurs when interactions cause energy loss in the incident primary electrons. Due to the energy loss, the scattered electrons have a longer wavelength than the incident primary electrons [72]. The properties discussed above can be used to characterize materials. 

The advantages of electron microscopy include a high resolution in the obtained images [72], which can vary depending on the selected technique [72]. These also include the range of magnification, among [10–500,000]× for scanning electron microscopy and [2000–1,000,000]× for transmission electron microscopy [72]. The use of an electron beam is another advantage since their interactions with the sample atoms provide information about the structural and chemical properties of the materials [72]. 

The disadvantages that accompany these techniques include electron-optical aberrations in the image formation, which limit the resolution of the image obtained [74]. The spherical aberration results from the inhomogeneity of the lens field over the rays in the off-axis [74]. Chromatic aberration occurs from the variation of the focal length of the electrons according to their energy [74]. Astigmatism is caused by a lack of symmetry in the lens, in its cylindrical shape, causing the intersection of the beams in different image planes [74]. Another disadvantage is the inherent interaction of electrons with air molecules, causing some of them to scatter on their way to the sample [72]; this can be solved by ensuring a vacuum environment in the sample, which can be low (0.1–10^−4^ Pa) or high (10^−4^–10^−7^ Pa) [72]. However, this environment can change the nature of samples with high vapor pressures by releasing water and volatile content [72]. The applied energy for the electron beam over the samples may cause some changes in the sample at the atomic level [72]. This is the case in Raman spectroscopy, where very low laser power prevents phase changes during measurements [12]. The introduction of electron-based techniques—including TEM and SEM—follows.

##### Transmission Electron Microscopy (TEM)

This technique enables the measurement of the morphology, structure, and chemistry of particles on a nanometric scale [75]. The electron beam, accelerated by an electron gun with a voltage between 80 and 300 kV [8,10,14,24,68,72], is spread and applied over a selected specimen area, and the transmitted electrons are collected by a detector located below the specimen, forming an image. A lower energy (i.e., <100 kV) should be used for lighter elements such as carbon to minimize specimen damages. The magnification of TEM ranges from 2 kX to 1000 kX. 

Various operation modes have been developed to maximize the information obtained from the specimen. Bright-field (BF) imaging is the most common TEM mode. As noted, electrons may scatter as they pass through the specimen, and microstructural features promote this scattering, such as increased thickness, increased mass, and grain boundaries. The capture of unscattered electrons forms a BF image, and the free scattered electrons produce a darker contrast in a BF image. On the other hand, capturing the scattered electrons forms a dark field (DF) image. DF imaging maps regions of the specimen that generate electron scattering. Electrons incoherently scattered at very high angles are also collected and form high angle annular dark field images (HAADF), which show strong contrast changes due to changes in atomic mass. This can be used to analyze the chemical composition of specimens [55,72].

**Figure 6:**
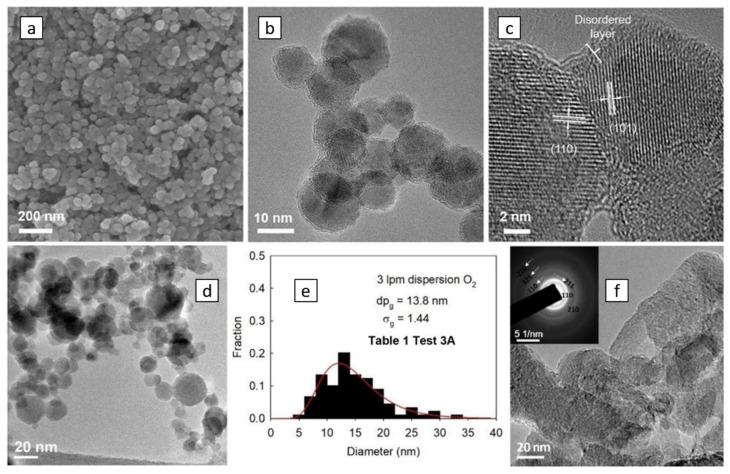
(**a**) FESEM, (**b**) TEM, (**c**) HRTEM techniques, for analysis of morphology of titanium-based NPs [55], reproduced with permission from Wu, S., et al., Small Methods; published by Wiley Online Library, 2020. (**d**) TEM image of flame-synthesized alumina nanoparticles [18], and (**e**) its size distribution, where $d_{\mathrm{pg}}$ is mean particle size and $\sigma_{g}$ is geometric standard deviation [18], Reproduced with permission from Okonkwo, O., et al., Journal of the American Ceramic Society; published by The American Ceramic Society, 2021. (**f**) TEM image of iron-oxide NPs, including SAED pattern at the top-left position for detection of hematite phase [11], reproduced with permission from Hong, H., et al., Proceedings of the Combustion Institute; published by Elsevier, 2019.

To generate meaningful images of high magnification, i.e., >400 kX, high-resolution TEM (HRTEM) is required [7,10,55,62]. This method requires that the specimens be ultrathin, i.e., in the order of 100 nm thickness or less. In this condition, electrons interacting with the crystal lattice diffract, forming interference patterns corresponding to atom positions. As a result, the lattice spacing of specimens, which can be used to identify the chemical composition, e.g., graphitization of carbon [10,62,76], is visible. Wu et al. [55] investigated the morphology of titania nanoparticles using the HRTEM (as shown in Figure 6c), where a well-ordered core contrasted with the disordered layer found at the surface of these NPs.

Another technique associated with TEM is the selected area electron diffraction (SAED), which provides information about the nanostructure of the specimen. The technique employs the principle of Bragg’s law, which gives the condition for constructive interference when incident electrons diffract from the atomic spacing of a specimen. As a result, a diffraction pattern (which could be bright spots or rings) is formed and imaged [77]. Measurements of the distance between spots/rings, their origin, and intensities provide the lattice spacing and the crystallinity of the specimen. Hong et al. [11] applied this method for the detection of structural phases in the iron-oxide NPs produced (as exhibited in Figure 6f), where the pattern obtained matched the features of the hematite phase. SAED is similar to X-ray diffraction, except that SAED is conducted within TEM, enabling the examination of nanoscale areas.

Over the years, variants of TEM have been developed to satisfy various requirements. To obtain images with better contrast and improved elemental maps, energy-filtered TEM (EFTEM), which allows electrons of specific energy to be detected, can be used [7,77]. Ismail et al. [7] confirmed the silica coating in titanium-based NPs using this technique and located the distribution of oxygen and titanium elements in the sample. Environmental TEM (ETEM) also allows the evolution of a particle under various conditions to be observed in real time, enhancing understanding of the effect of the surroundings on particle growth.

TEM images are post-processed with software to obtain more specific information (e.g., particle sizes and lattice spacing [4,18]). Figure 6e displays particle size distribution obtained from a TEM image of alumina NPs (Figure 6d), where small particle sizes dominate, demonstrating that the gas-to-particle formation governs the synthesis of these particles [18]. However, analyses using TEM images often suffer from user bias, as users tend to select large particles or obvious targets. Moreover, as observed by Ismail et al. [10], each nanoparticle possesses its unique crystal orientation for titanium-based NPs, and dispersion in the measured sizes can occur from the different trajectories that the particles take in the flame flow field, as shown in the synthesis of alumina NPs [18]. Thus, to obtain statistically significant results, a minimum of eight TEM images and five hundred particles [15] are recommended.

##### Scanning Electron Microscopy (SEM)

SEM is useful for the analysis of the sample surface. The incident beam of electrons is concentrated in one point over the surface of the specimen; this point is then moved uniformly over the entire surface [72,78,79]. Unlike TEM, electrons in SEM possess low energy, with accelerating voltage ranging from 1 to 30 kV [4,17,72,78]. The magnification of SEM can range from 10 to 500 kX. Detecting secondary electron emission forms SEM images when the incident electrons hit the specimen, revealing the surface topography of the nanoparticles [11,25,26,80].

Because of the limitations of the electron source of SEM, its effective spatial resolution is around 10 nm [72]. Field emission SEM (FESEM) generates electrons using the field emission concept, which is recommended to achieve a higher resolution [79]. FESEM is widely used to analyze the morphology and size of flame-synthesized nanoparticles [3,4,9,13,24,55,68,76]. Wu et al. [55] determined the particle sizes of titania NPs between 10–20 nm and proved their agglomeration using this technique, as seen in Figure 6a. Ultra-high resolution, i.e., 0.5 to 2 nm, can be achieved by helium ion microscopy (HIM) [81]. Using helium ions, which have higher brightness and a larger momentum than electrons, images of a higher resolution with a larger depth of field can be achieved [82]. HIM provided images of flame-generated nanoparticles with a size of less than 2 nm [83,84].

Along with SEs, A SEM microscope can also detect the BSE signal, and BSE images are formed, providing information about the chemical composition of specimens. Because the BSE signal is proportional to the atomic number of an element, the brighter areas of a BSE image represent heavier elements. Assessing the contrast of BSE images provided the distribution of two or more phases with significantly different atomic masses. 

All materials emit X-rays when bombarded by incident electrons. X-rays of different energies are collected to form an energy-dispersive X-ray (EDX) spectrum [72]. Since each element has its unique EDX spectrum, the elemental components of specimens can be identified [17,24,68,80]. Han et al. [80] obtained an elemental mapping of synthesized CNTs which contained iron-oxide NPs, proving a higher concentration of iron at the tip of the CNTs, and a more uniform distribution of carbon along this structure.

##### Atomic Force Microscopy (AFM)

AFM is a technique used for characterizing material surfaces. A probing tip is attached to a cantilever-type spring, which deflects in response to the force between the probing tip and the sample surface. A deflection sensor records the motions from the order of $10^{-6}$ to $10^{-10}$ m [85]. The sample is scanned relative to the probing tip, obtaining a high-resolution, three-dimensional image containing surface features on an atomic scale. Because of the tiny force between the probing tip and the sample, i.e., from $10^{-11}$ to $10^{-6}$ N, the structure of molecules (e.g., the order of $10^{-9}$ N for a covalent bond) can hardly be destroyed [85]. Thus, the technique is considered non-destructive. Unlike its precursor, scanning tunnel microscopy (STM), AFM is also useful for non-conductive materials [85].

Because of its versatility, AFM has been widely used in nanoparticle research to reveal the three-dimensional topological map of nanoparticles. Like other imaging techniques, AFM gives information on the morphology of nanoparticles, i.e., the degree of aggregation and the change in shape and size [86,87,88,89,90]. The size distributions obtained by AFM are consistent with techniques such as TEM and dynamic light scattering (DLS) [91]. In addition, AFM can determine the size distribution of mixed samples of different particle sizes [91]. Because of its three-dimensional feature, AFM also gives the measurement of particle heights [86,88,90], which is remarkable because it provides clues for the physical properties of nanoparticles. For example, combustion researchers used AFM to identify nascent soot, which flattens as it hits a sampling grid [86,92,93]. The observation suggested that nascent soot, which is not detectable by optical techniques, is liquid-like and far from being mature [86]. On the other hand, AFM can be used to select and manipulate specific nanoparticles on a surface [94,95].

AFM measurements are affected by deposition methods and substrates for nanoparticle deposition. Deposition methods may skew the size distributions of nanoparticles [91,96]. If the nanoparticles are over-diluted for deposition, very few are deposited on a substrate and vice versa [96]. In addition, because AFM relies on the movement of the probe tip, the surface roughness of a substrate also contributes to the error in size measurements [87]. Delvallee et al. [87] recommended that mica sheets and silicon wafers, which have relatively lower roughness, should be used for AFM. However, mica is hydrophilic and may lead to the agglomeration of nanoparticles, producing measurement errors [96]. Thus, substrates with higher hydrophobicity can solve this problem. Moreover, artifacts are created as the probe tip does not necessarily follow the outer edge of a particle [86,87,97]. Therefore, AFM should be used with other techniques, such as TEM and SEM, to reliably estimate particle sizes.

#### 4.2.2. The Brunauer-Emmet-Teller Method (BET)

The BET is the most common technique used to determine the specific surface area (SSA) of nanoparticles. This variable represents the surface area of solid nanoparticles per unit of mass [98,99]. The BET method enables the SSA value calculation based on the volume of N_2_ gas adsorbed by the nanoparticles at 77 K [8,10,18,55,100]. Assumptions include the N contact with the entire surface of the nanoparticles and their infinite number of layers [100]; hence, the adsorption occurs without interaction among the layers. 

The equivalent BET particle size (d_BET_) can be derived from the calculated SSA [7,14,18], using Equation (1), obtained from the study of Rubio et al. [14]:

(1) $$d_{\mathrm{BET}}=\frac{6000}{\mathrm{SSA}*\rho }$$

where d_BET_ is expressed in [nm], SSA is in [m^2^/g], and ρ is the density of nanoparticles [g/cm^3^], which is not the same value as the bulk density. The equation assumes that the particles are monodispersed and spherical. The study of Wu et al. [55] determined a higher SSA for the flame-synthesized titania NPs than with that measured in the commercial P25 type.

#### 4.2.3. Dynamic Light Scattering (DLS)

Dynamic light scattering (DLS) provides particle size distribution profiles. Samples for DLS are prepared by suspending nanoparticles in a liquid. A laser beam passes through the sample, and the scattered light intensity associated with the Brownian motion of particles is measured at a given scattering angle for a given period [101,102,103]. The resultant measurements are then converted into size distribution profiles. It is worth noting that DLS gives hydrodynamic diameter, derived from the Stoke-Einstein equation, and is a function of the measured particle diffusion coefficient. For non-spherical particles, the hydrodynamic diameter is not the same as the actual geometric diameter measured by other techniques, e.g., TEM [16,68].

The benefits of DLS include fast acquisition, ease of use, and the possibility of reusing sample particles. However, the interpretation of DLS measurements is subject to factors such as suspension concentration, particle size, polydispersity, and surface property. This technique also provides information about the surface charge in NPs, as shown by Chang et al. [68], for CNPs produced by flame synthesis. Although flame synthesis studies mostly applied this method in ex-situ conditions, it has been applied for in-situ measurements of nanoparticle sizes [104,105,106].

#### 4.2.4. X-ray Diffraction (XRD)

The application of the X-ray diffraction (XRD) technique reveals the structure of nanoparticles [7,8,9,10,11,12,13,15,16,17,25,26,27,55,68,80]. The working principle (i.e., the basis of Bragg’s law) is the same as SAED, except that X-ray generated by CuK_ radiation is the radiation source. Diffracted radiation from a sample is measured at different angles to produce the XRD spectra. Peaks in an XRD spectrum indicate lattice planes of the sample, enabling identification of the sample’s chemical composition.

Unlike crystalline materials, amorphous materials yield broadened peaks, indicating structural strains, stacking faults, dislocations, and defects [107,108,109]. Thus, the width of the peak correlates with the disorder of the nanostructure of the materials [110,111,112]. After the targeted peak width is known, the use of the Scherrer equation [113] (shown in Equation (2)) allows the estimation of the size of the lattice plane of the corresponding peak:

(2) $$B\left(2\theta \right)=\frac{K\lambda }{\mathrm{Lcos}\theta }$$

where B is the FWHM (full-width at half-maximum) of a peak at 2θ, L is the lattice plane length, λ is the wavelength of the incident X-ray, and K is the shape factor, which varies from 0.62 to 2.08. Other information for nanostructures, such as interlayer spacing (Bragg’s equation) [113], and phase composition (e.g., aromaticity for carbon materials [114]), can also be estimated from the XRD spectrum. Measuring the peak width can reveal the degree of graphitization [110,111,115,116] and interlayer spacing [113,117] of carbonaceous materials. Rietveld analysis provides detailed and precise information on the XRD spectra [118]. It should be noted that a relatively large number of samples is necessary for reliable measurements. However, the latest technology allows XRD measurement of thin films [119].

**Figure 7:**
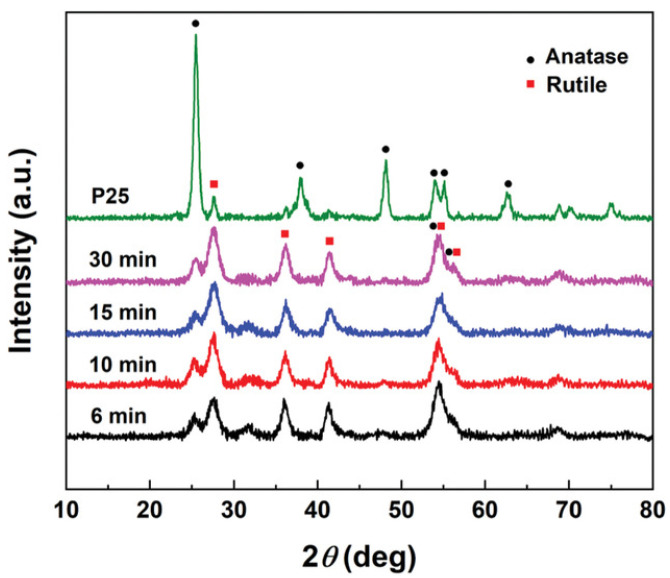
XRD patterns for titania NPs obtained at various levels of deposition time, compared with the pattern obtained for the commercial P25 TiO_2_ [55]. Reproduced with permission from Wu, S., et al., Small Methods; published by Wiley Online Library, 2020.

Ismail et al. [7] applied this technique to determine the effect of adding iron, silicon, and vanadium elements in the precursors over the composition of structural phases in the flame-made titania NPs using this technique. Their results exposed the dominance of the anatase phase with a small percentage of carbon in the precursors; this content decreased with high carbon contents. Moreover, adding vanadium made the rutile phase dominant [7]. Figure 7 displays the XRD patterns obtained for flame-synthesized titania NPs at different deposition times and includes the pattern of the P25 TiO_2_ for their comparison, identifying the characteristic peaks of anatase and rutile phases in each pattern [55].

#### 4.2.5. X-ray Photoelectron Spectroscopy (XPS)

This quantitative method determines the elemental composition at the surface of a sample [120,121], and it is known for characterizing the surface chemical state of flame-synthesized materials [7,8,9,11,14,16,27,55,68,76]. The X-ray beam irradiation over the sample quantifies parallelly the kinetic energy and the number of electrons leaving [121]. Moreover, this method is surface-sensitive [55] since this technique analyzes the material in a range of 10 nm from the top [121]. Ultra-high vacuum conditions (UHV) are required to perform this method [120,121,122].

With this method, a commonly observed feature in the analysis of nanoparticles is the presence of oxygen peaks [14,68], which are caused by partial oxidation in the synthesis of the NPs [76]. This feature also indicates that the NPs contained oxygen because of the availability of defect sites—often called oxygen vacancies (OVs), which may provide adsorption features to NPs [68] and can also be present to maintain the charge equilibrium [9,55]. The study of Ismail et al. [7] obtained detailed information about the XPS spectra for the C 1s, Ti 2p, Fe 2p, Si 2p, O 1s, and V 2p core levels. This high-resolution data provided the chemical composition of the samples and demonstrated the presence of SiO_2_ on the surface of the titanium-oxide NPs, corroborating the findings of the EFTEM performance in these produced particles (see Figure 2g) [7]. The XPS spectra also asserted the existence of doped vanadium in the TiO_2_ nanoparticles.

#### 4.2.6. Raman Spectroscopy

Raman spectroscopy provides information about chemical structure, phase, crystallinity, and molecular interaction. The working principle is based on light scattering from the molecules of samples. While most light scatters away at the same wavelength (i.e., Rayleigh scattering), when a laser shines on a sample, a small amount of light is scattered at different wavelengths (i.e., Raman scattering [123,124]). Changes in wavelength depend on the chemical structure of the sample. A Raman spectrum was constructed by comparing these changes (i.e., Raman shift). Each peak on the spectrum represents a specific chemical bond (e.g., C-C). While highly structured materials show sharp and distinct peaks, amorphous materials (e.g., carbon black) exhibit line broadening, making deconvolution of peaks a requirement for spectrum interpretation.

This method provided information at the surface of samples within a depth range from 30 to 60 nm from the top [125]. Raman spectroscopy performance requires a shorter time than techniques such as XRD, which has a long measurement time [12]. It is worth noting that although ex situ measurements using this technique are typical, in situ applications have also been found [126,127,128,129].

For nanoparticle synthesis, Raman spectroscopy is used to identify structural phases, such as hematite in Fe_x_O_y_ [11,12,13] and anatase in the TiO nanoparticles [7,55]. For carbon-based materials (e.g., carbon black and CNTs), this method evaluates the degree of their structural disorder [3,4,7,11,24,26,68,76,80], using the peaks defined for the D and G bands. This technique is useful for detecting and studying the presence of oxygen vacancies (OVs) in nanoparticles [16,17]. The results from Ismail et al. [7] confirm the dominance of the anatase phase in all titania samples—except for the material containing vanadium, where the rutile phase content is the highest—which was in agreement with the XRD results. Moreover, the higher peaks for the D and G bands are notable for the samples with iron content [7].

#### 4.2.7. Fourier-Transform Infrared (FTIR) Spectroscopy

Studies using the Fourier-transform infrared (FTIR) spectroscopy method identify compounds in a mixture sample. It measures the absorbed radiation from a sample. Infrared radiation (10,000–100 cm^−1^) passes through the sample via an interferometer, which modulates the wavelength from a broadband infrared source. The sample absorbs part of the energy and converts it into vibrational energy in the molecular bonds, producing an infrared absorption spectrum, which is unique for each molecular bond [130,131]. Thus, by comparing the reference spectrum library, the chemical species can be identified.

**Figure 8:**
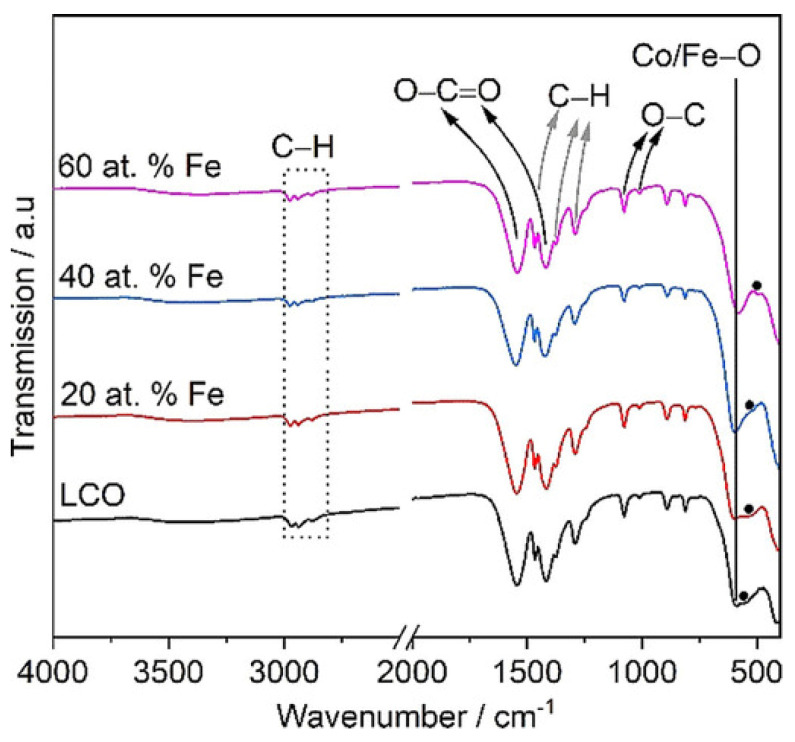
FTIR spectra of perovskite nanoparticles, exposing the C-H, O-C=O, O-C, and Co/Fe-O functional groups. Dots represent a secondary band related to the last functional group [27]. Reproduced with permission from Alkan, B., et al., ChemElectroChem; published by Chemistry Europe, 2019.

FTIR is used in the community to identify nanoparticle functional groups [6,27,68]. For example, Mohapatra et al. [6] used FTIR to show that onion-like carbon is highly dispersible and stable over time because of surface functional groups, which are carboxylic and hydroxyl functions. Alkan et al. [27] performed this technique for analysis of the functional groups present in the perovskite NPs produced in flame-assisted pyrolysis; the obtained spectra are plotted in Figure 8. Their results agreed with those from previous studies; however, the iron content variation in the precursor modified the nature of Co-O bonds in the NPs [27]. It is worth noting that although FTIR is classified as an *ex-situ* method, various combustion studies detected gas-phase species (e.g., NO, N_2_O, and CO_2_) in flames using this method [132,133,134,135].

#### 4.2.8. Photoluminescence Spectroscopy (PL Spectroscopy)

Photoluminescence (PL) spectroscopy measures the emission of photons from matter when stimulated by incident light, which has higher energy than the energy bandgap of the matter [136]. The excited electrons return to the ground state and emit photons in parallel. PL is used to detect impurities and defects and evaluate bandgap energy, molecular structure, and nanoparticle crystallinity. 

By studying PL spectra at different conditions (i.e., temperature and excitation power), Zeferino et al. [137] assessed the role of gold in gold-doped ZnO nanoparticles. The accumulation of gold distorted the ZnO lattice and altered the Pl properties of ZnO nanoparticles. Furthermore, Wu et al. [55] used time-resolved PL spectroscopy to assess the photophysical properties of flame-made TiO_2_. They found that flame-made TiO_2_ exhibited photocatalytic activity superior to commercially available TiO_2_ because the intensity of the former decayed faster than the latter in the PL measurements.

#### 4.2.9. Thermogravimetric Analysis (TGA)

This technique measures the mass of a sample over time with temperature change. This measurement provides information about physical and chemical changes (i.e., thermal stability [68]) in the sample with the temperature variation. It can also determine the purity and the content of volatile or carbonaceous matter in a sample [7,13]. The effectiveness of catalytic reactions of nanoparticles can also be assessed using TGA. Ismail et al. [7] found that iron-based TiO_2_ achieved complete oxidation at a lower temperature than TiO_2_ without iron.

**Figure 9:**
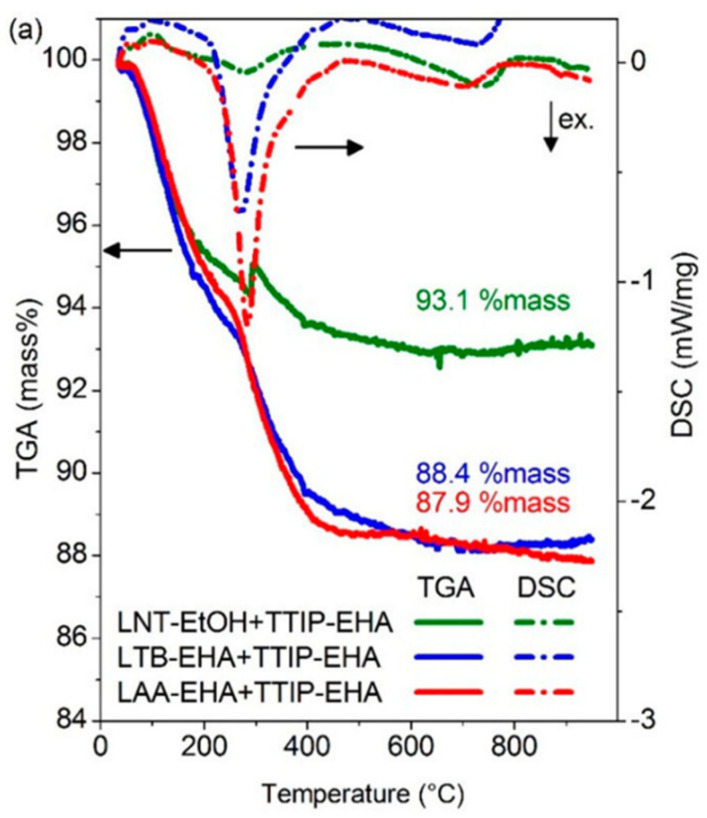
Results obtained from the coupling of TGA and DSC analysis, carried out in Li_4_Ti_5_O_12_ particles produced through flame-spray pyrolysis (FSP), using various precursors and solvents [62]. Reproduced with permission from Meierhofer, F., et al., ACS Applied Materials & Interfaces; published by ACS publications, 2017.

TGA may be combined with other techniques to carry out more detailed analysis. For example, Carvajal et al. [13] combined TGA with a gas analyzer and a mass spectrometer (MS) to identify thermal events when heating Fe_x_O_y_ nanoparticles in TGA. Gas emission helped the authors identify the events of water vaporization, solvent decomposition, and oxidation of carbonaceous materials [13]. Meierhofer et al. [62] coupled TGA with differential scanning calorimetry (DSC) and a mass spectrometer (MS) to evaluate purity and identify volatile compounds evolving in the samples. Figure 9 displays the results from this TGA and DSC coupling.

#### 4.2.10. UV-vis-NIR Spectrophotometry

Ultraviolet-visible-near infrared (UV-vis-NIR) spectrophotometry measures the radiation absorption of samples at wavelengths between 200 and 2500 nm [138]. The absorption intensity is species-dependent; therefore, it is possible to determine the presence of a substance in a sample. Subsequently, material properties could be assessed. In particular, it is useful to assess the properties of nanotubes as they are active in the UV-vis-NIR region. 

**Figure 10:**
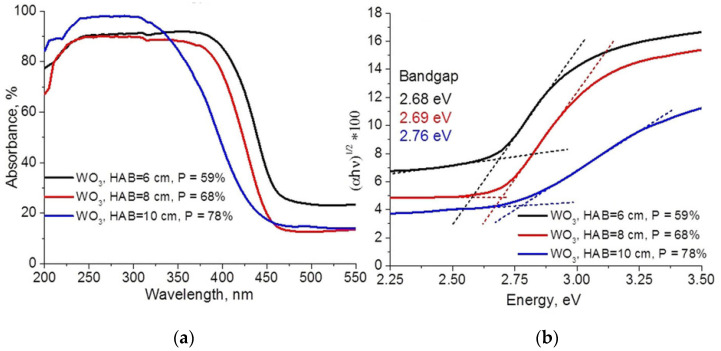
(**a**) UV/Vis spectra obtained for tungsten-oxide NPs collected at different heights above burner (HABs). (**b**) Tauc plots obtained for the same samples [17]. Reproduced with permission from Chen, H., et al., ChemPlusChem; published by Chemistry Europe, 2018.

Ryabenko et al. [139] used the technique to evaluate the effectiveness of different purification processes for soot-containing single-wall carbon nanotubes. By comparing the absorption spectra, the authors concluded that centrifugation was the most effective purification method. Chang et al. [68] used this method to identify the absorptivity of a cationic dye of carbon nanoparticles derived from corn oil; they doped the CNP into the dye, which absorbs UV-vis radiation. As the absorption intensity decreased with the increasing concentration of the CNP, the study concluded that this type of CNP absorbs the cationic dye. Figure 10 shows an example of the UV/vis spectra for WO_3_ samples, taken from the study of Chen et al. [17], where the plotted Tauc plots (Figure 10b) exposed information about the bandgap of the samples. The authors concluded that the variation in height above the burner—and consequently the temperature—affected the optical features in the synthesized samples [17].

#### 4.2.11. Vibrating Sample Magnetometer (VSM)

A vibrating sample magnetometer quantifies the magnetic properties of nanoparticles. The working principle consists of a constant magnetic field applied over the sample. As the magnetized sample started vibrating, an electric field was induced, which is proportional to the degree of magnetization. Repeating this process with different magnitudes of the constant magnetic field exposes the magnetic properties of the sample. 

The performance of VSM exhibited the magnetic properties of iron-based NPs (i.e., Fe_x_O_y_) [13,140,141]. Because of their superparamagnetic nature (i.e., size-dependent magnetization), it was important to understand their magnetic properties. The magnetization of these NPs increased with particle size and magnetite content [13].

### 4.3. In-Situ Measurement Techniques

For a better understanding of nanoparticle formation, accurate and detailed spatial and temporal characterization of nanoparticles was necessary, using both *in-situ* and *ex-situ* techniques. 

#### 4.3.1. SMPS—Scanning Mobility Particle Size

The scanning mobility particle sizer is based on the mobility of a positively charged particle in an electric field. The instrument usually consists of a neutralizer (bipolar charger), a differential mobility analyzer (DMA), and a condensation particle counter. The neutralizer charges the collected particles before entering the DMA for particle sizing. In the DMA, a negatively charged rod attracts the positively charged particles, which travel through the sheath gas at rates determined by their electrical mobility. Particles of a given mobility enter the condensation particle counter, yielding particle size distribution spectra. Because the sizes are determined based on the electrical mobility of particles, SMPS yields mobility sizes [142,143], although the difference between mobility sizes and actual geometric sizes is small [144]. Conventional SMPS spectrometers lack the resolution for the analysis of nanoparticles. For nanoparticle applications, SMPS spectrometers equipped with high-resolution DMAs [145], such as nano-DMA [84], Vienna DMA [146,147], and radial DMA [148,149], are required. 

Relating electrical mobility to size is challenging. The semi-empirical Stokes-Milliken equation provides satisfactory results for coarse particles or the continuum region (Kn << 1) [150,151]. However, for finer particles or the free molecule region (Kn >> 1), the Stokes-Milliken equation is insufficient as factors such as diffuse/specular scattering and potential interaction become non-negligible [151]. As such, different researchers proposed corrections to the Stokes-Milliken equation to predict nanoparticles [152,153,154]. Ehn et al. [155] reported that the modified Stokes-Milliken equations [152,153] worked well down to the size of 1 nm. Non-spherical shapes give complexity to the analysis of electrical mobility. The Stokes-Milliken equation with a modified Knudsen number was proposed for non-spherical particles in the transition and free molecular regimes. However, it should be solved numerically [156].

**Figure 11:**
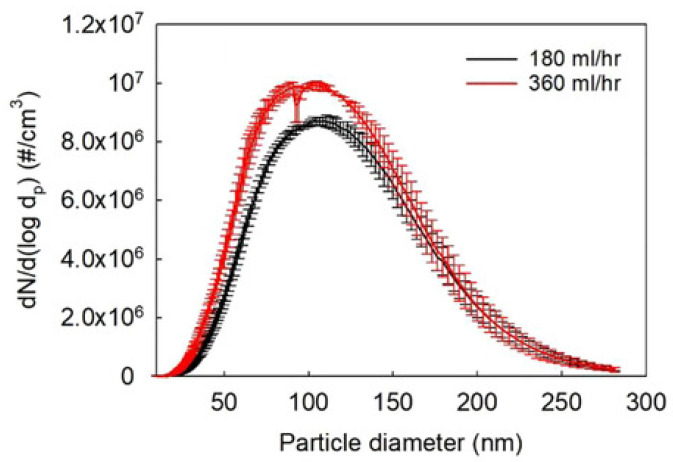
Particle size distribution of alumina NPs produced through flame synthesis at different precursor feed flow rates [18]. Reproduced with permission from Okonkwo, O., et al., Journal of the American Ceramic Society; published by The American Ceramic Society, 2021.

When sampling particles for online SMPS, samples should be properly diluted with nitrogen, and the pressure difference must be carefully controlled to minimize particle loss in the sampling line due to particle coagulation and wall diffusion [157,158]. Without dilution, particle loss is about 10% in only 20 ms [158]. It is essential to dilute the sample to a critical level so that the resultant particle size distribution is independent of the dilution ratio [158]. Okonkwo et al. [18] obtained the particle size distribution using this technique; the measured distributions at different precursor flow rates are plotted in Figure 11. The dominant growth mechanism is the particle collision, demonstrated by the similar standard deviation determined at all conditions [18]. The larger geometric mean diameter, measured through SMPS and compared with that determined from TEM and BET methods, confirmed the agglomeration of particles [18].

#### 4.3.2. Laser Diagnostic Techniques

**Figure 12:**
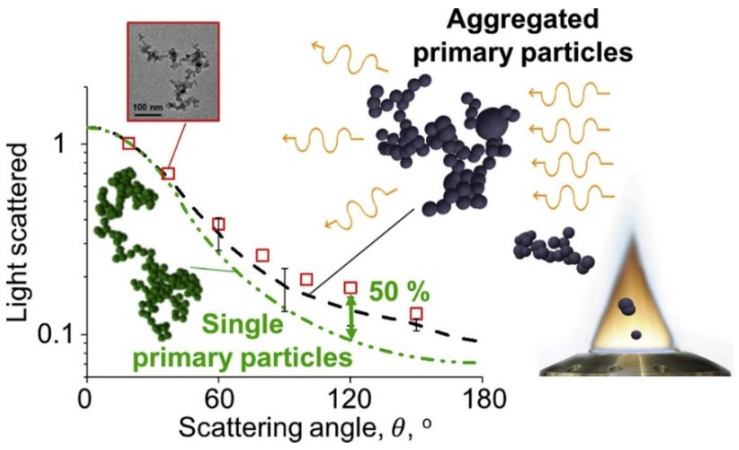
Functions of light scattering signal with scattering angle for single primary particles and aggregated primary particles [169]. Reproduced with permission from Kelesidis, G.A., et al., Powder Technology; published by Elsevier, 2020.

The application of light-induced scattering techniques proved the existence of nascent soot, particle size, mass fractal dimension, aggregate size, and number of primary particles per aggregate [159,160,161,162,163,164,165,166,167,168]. Generally, the laser beam is focused first on a point in the probe volume with a diameter near 1 mm; the scattered light is detected by a photomultiplier tube. Visible-light scattering is the most common technique, compared with the techniques using visible/UV light, microwaves, near IR, X-rays, and neutrons. The intensity of the scattering signal is highly sensitive to the scattering angle, as shown in Figure 12 [169]; this allows the measurement of fractal characteristics by multi-angle light scattering techniques, which was used by Yang et al. [159] to investigate the fractal characteristics of the titania particles produced from a premixed flame aerosol reactor. A CW argon-ion laser was used to yield a 488 nm incident beam. Yang et al. [159] observed the isolated titania particles in the high-temperature region, and a sintering equation was developed based on the light scattering data. Simon et al. investigated the size distribution of TiO_2_ nanoparticles using a wide-angle light scattering method with pulsed light at 532 nm, which could also identify the presence of morphological fraction [170]. The formation of silica particles in co-annular diffusion flame was investigated by Adrian et al. [160] using an in situ small-angle X-ray scattering (SAXS) technique, with the ability to monitor the mass fractal dimension, aggregate size, and the number of primary particles per aggregate. Ceolato et al. [171] demonstrated the ability of picosecond short-range elastic backscatter LiDAR to quantify the carbon number and mass concentration.

The laser-induced incandescence (LII) technique consists of a laser beam heating the sampled particles, and the subsequent incandescence decay curve can determine the size of particles and their volume fraction [172,173,174]. For details of LII theory refer to the work by Michelsen et al. [175]. LII is widely used in the soot community to probe flame soot, as illustrated in the review paper of Schulz et al. [176], which does not sublimate until above 4000 K. However, the lack of knowledge from the material properties (including sublimation temperature, radiative properties, specific heat, among others) limits the application of LII on metal nanoparticle diagnosis. Iuliis et al. [177] explored the applicability of the LII method to TiO_2_ nanoparticles in flame spray synthesis. Their results showed that the signal spectrum was highly sensitive to laser fluence. At low laser fluence, the fluorescence signal of the anatase TiO_2_ nanoparticle can be detected; the incandescence signal appears at high laser fluence, and the breakdown signal appears when the laser fluence moves beyond a threshold value. Modelling the laser heat and the subsequent cooling process could help to interpret the LII signal from metal-based nanoparticles. Gurentsov et al. [178] obtained the size of ion nanoparticles by a combination of LII experiments and simulation. Daun et al. [179] measured the size distribution of silicon nanoparticles using time-resolved LII (TiRe-LII) measurements and simulation.

It should be noted that some small gas-phase particles present fluorescence characteristics [180]. Bruno et al. [181] detected the carbon particle with a diameter of 3.3 nm based on the laser-induced fluorescence (LIF) technique. 

Flame temperature is a crucial parameter controlling the quality of nanoparticles. The information on the emission spectrum of particles is useful for interpreting flame temperature [182,183]. Jenkins et al. [184] measured the flame temperature using the modulated absorption/emission pyrometry, and the uncertainty is within 20 K. Mekhrengin et al. [185] combined the soot pyrometry and C_2_* emission spectroscopy for flame temperature measurement. Zhong et al. [186] performed color ratio pyrometry to record the temperature evolution for synthesizing metal-oxide nanoparticles. The temperature measurement in synthesis flame may be more complicated. For example, De Iuliis et al. [182] demonstrated the non-isothermal nature of the emitting particles, i.e., the hot and cold particles coexist in the probe volume in a synthesis flame.

**Figure 13:**
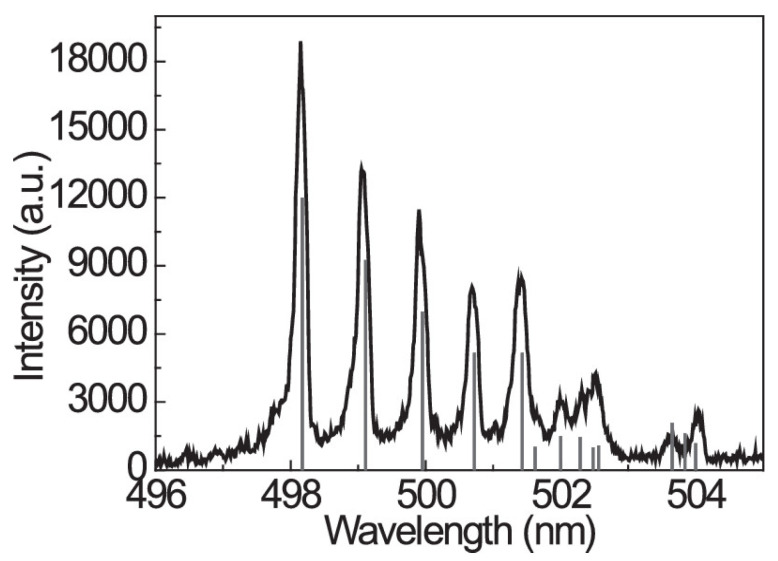
Spectra of emissions (around 500 nm) from PS-LIBS of titania nanoparticles 25 mm above the burner at a laser energy of 23 mJ/pulse. Lines correspond to the NIST database [191]. Reproduced with permission from Zhang, Y. et al., Applied Physics Letters; published by AIP Publishing, 2014.

**Figure 14:**
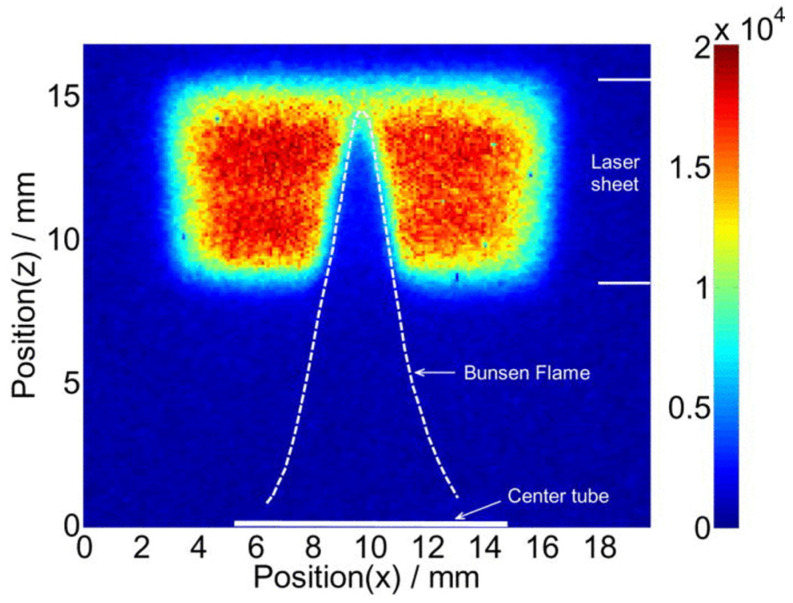
Two-dimensional distribution of particle-phase Ti imaged with a 500 nm bandpass filter, showing the rapid conversion from gas-phase precursor to TiO_2_ nanoparticles across the flame and conservation of particle volume fraction in the coagulation process downstream of the flame [191]. Reproduced with permission from Zhang, Y. et al., Applied Physics Letters; published by AIP Publishing, 2014.

Heated by high laser fluences, the nanoparticle can partially or entirely evaporate, offering the opportunity to monitor gas-to-particle conversion. This behavior is the working concept of laser-induced breakdown spectroscopy (LIBS). Amodeo et al. [187] demonstrated the feasibility of an online LIBS monitoring system for real-time composite nanoparticle measurements. Morgan et al. [188] explored nanosecond-duration LIBS for detecting airborne metals and concluded that the nanosecond-duration LIBS could detect AI at concentrations of 5%–16% by weight. Mukherjee et al. [189] determined the extent of oxidation and coating thickness of aluminum nanoparticles using LIBS. The breakdown only occurred when the nanoparticles were ablated at low laser fluence, making it possible to distinguish the elements from the solid particle phase. This technique is useful for understanding gas-particle conversion, and is termed a low intensity phase-selective laser-induced breakdown spectroscopy (PS-LIBS) [190]. The emission spectra of titania nanoparticles by PS-LIBS is illustrated in Figure 13, taken from the study by Zhang et al. [191] which also obtained two-dimensional images of gas/particle phase transition of metal oxides in high temperature flow conditions using PS-LIBS, as shown in Figure 14.

## 5. Conclusions

This review explored the latest findings in the formation, diagnosis, and applications of carbon and metal-oxide nanoparticles synthesized in flame. Specifically discussed were the types of burners used in flame synthesis for some laminar flames at small scales. The counterflow non-premixed flame enabled practical measurements and prediction of the combustion parameters, allowing the study of the effects of these parameters on NP properties. The multiple diffusion flame setup provided the benefit of a premixed flame in a safer operation environment, allowing the scale-up of this technology for industrial applications. This last configuration also enabled the use of liquid precursors for flame-assisted pyrolysis, broadening the applications to metal-oxide nanoparticles. The effects of operating parameters—including equivalence ratio, flame temperature, fuel type, and substrate materials—on particle size distribution, morphology, and chemical and structural composition were examined. The equivalence ratio influences the flame temperature in premixed flames, and the stoichiometric mixture fraction affects this temperature in diffusion flame configurations. The flame temperature and the residence time exerted the main influence over the properties of the synthesized particles since these parameters dominate the sintering process for the formation of metal-oxide nanoparticles and the particle nucleation and size growth modes in the synthesis of carbon-based nanoparticles. The stability of the structural phases of metal-oxide NPs is the reason for the dominance of the flame temperature over the structural phase composition of these materials. This review introduced various in situ and ex situ measurement methods for detecting particle properties and discussed the possible limitations of each technique. Nevertheless, a knowledge gap exists regarding the reaction pathways at the molecular scale of these metal-oxide and carbon nanoparticles, and therefore needs further extensive investigation.

## Figures and Tables

**Table 1:** Summary of flame configurations and the burner geometry commonly used for the study of flame synthesis of carbon and metal-oxide nanoparticles.

Flame Configuration	Burner Geometry	Produced Nanoparticles	Reference
Premixed flames	Bunsen	CNPs	[29]
	Multi-element diffusion burner	SiO_2_	[30]
Diffusion flames	Normal/Inverse coflow	Fe_x_O_y_	[31]
		TiO_2_	[32]
	Normal coflow	CNTs	[33]
	Inverse coflow	CNP—soot	[34]
		CNTs	[35,36]
	Counterflow	CNTs	[33,37]
		Fe_x_O_y_; CNP—soot	[38]
	Multi-element diffusion burner	SiO_2_	[30]
		TiO_2_	[39]
Flame spray pyrolysis (FSP)	Diffusion atomizing burner	Pt	[40,41]
		Al_x_O_y_, Zr_x_O_y_, Mn_x_O_y_, YSZ	[42]
		Al_x_O_y_	[43]
	Premixed atomizing burner	NCMs	[44]
		Pt/TiO_2_	[45]
		SiO_2_	[46]

**Table 2:** Comparison of the combustion parameters analyzed in nanoparticles flame-synthesis, considering the influence over the particle properties.

Combustion Parameter	Flame Configuration	Produced NP	Particle Properties	Reference
Equivalence ratio	Premixed flame	SiO_2_	Number density, particle size,	[15]
		TiO_2_	Morphology	[55]
	Flame spray pyrolysis	Fe_x_O_y_	Number density, structural phase composition	[13]
			Particle size, structural phase composition	[12]
		Al_x_O_y_	Particle size	[18]
Stoichiometric mixture fraction	Diffusion flame	CNP—soot	Number density	[56]
		Fe_x_O_y_	Structural phase composition	
Flame temperature	Premixed flame	CNP	Particle size, mass yield, morphology	[57,58]
		CNT	Morphology	[26]
		TiO_2_	Structural phase composition	[10,59]
		SiO_2_	Structural phase composition	[15]
		Fe_x_O_y_	Mass yield	[60]
	Diffusion flame	Fe_x_O_y_	Particle size, structural phase composition	[56]
		SiO_2_, TiO_2_	Particle size	[61]
	Flame spray pyrolysis	SiO_2_	Morphology	[14]
		Fe_x_O_y_	Morphology, structural phase composition	[18]
		Li_x_Ti_y_O_z_	Morphology	[62]
Fuel type and precursors	Diffusion flame	CNP—soot	Number density	[25]
	Flame spray pyrolysis	Fe_x_O_y_	Morphology	[12]
			Particle size, number density	[18]
			Number density	[13]
		Li_x_Ti_y_O_z_	Morphology	[62]
Residence time	Premixed flame	TiO_2_	Particle size, structural phase composition	[8]
			Morphology, structural phase composition	[10]
		CNT	Morphology	[26]
	Flame spray pyrolysis	Fe_x_O_y_	Morphology, structural phase composition	[18]
			Particle size	[12]
			Structural phase composition	[13]
Substrate material	Diffusion flame	CNT	Number density	[25]
			Morphology, number density	[3]
		Fe_x_O_y_	Number density	[11]
Flame configuration	Diffusion flame	CNT	Particle size	[25]
	Premixed flame	SiO_2_		[15]
	Flame spray pyrolysis	Li_x_Ti_y_O_z_		[62]
		Perovskites (Fe_x_O_y_)		[27]
		Al_x_O_y_		[18]

## Data Availability

Not applicable.

## Nomenclature

**Abbreviature**	**Definition**
a.u.	Arbitrary unit
ACL	Amorphous carbon layer
AFM	Atomic force microscopy
Al_x_O_y_	Aluminum oxide
α-Al_2_O_3_	Corundum phase—Aluminum oxide
γ-, η-, ρ-, θ-, δ-, χ-, κ-Al_2_O_3_	Metastable phases—Aluminum oxide
Ar	Argon
BET	Brunauer-Emmet-Teller method
BF-TEM	Bright field—transmission electron microscopy
BSE	Backscattered electrons
°C	Celsius degree (temperature unit)
cm^3^	Cubic centimeters (volume unit)
CNO	Carbon nano—onions
CNP	Carbon nanoparticles
CNT	Carbon nanotubes
CO_2_	Carbon dioxide
CuK	Cooper-Potassium
CVD	Chemical vapor deposition
CW	Continuous wave
C_X_H_Y_	Hydrocarbon compounds
Deg.	Degree (Angular position unit)
DF-TEM	Dark field–transmission electron microscopy
DLS	Dynamic light scattering
DMA	Differential mobility analyzer
DSC	Differential scanning calorimetry
EDX	Energy dispersive X-ray (spectrum)
EFTEM	Energy-filtered transmission electron microscopy
ENM	Engineered nanomaterials
ETEM	Environmental transmission electron microscopy
FCCVD	Floating catalysts–chemical vapor deposition
Fe(II)	Iron (+2 oxidation state)
Fe(III)	Iron (+3 oxidation state)
FeO	Wustite phase–iron oxide
FESEM	Field emission–scanning electron microscopy
Fe_x_O_y_	Iron-oxide
α-Fe_2_O_3_	Hematite phase–iron oxide
γ-Fe_2_O_3_	Maghemite phase-iron oxide
Fe_3_O_4_	Magnetite phase-Iron oxide
FSP	Flame spray pyrolisis
FTIR	Fourier-transform infrared
FWHM	Full width at half maximum
HAADF	High-angle annular dark field
HIM	Helium ion microscopy
hr	Hour (time unit)
HRTEM	High resolution-transmission electron microscopy
I_D_	Signal intensity of the D-band (Raman spectroscopy)
I_G_	Signal intensity of the G-band (Raman spectroscopy)
K	Kelvin (temperature unit)
kV	Kilovolts (electric potential difference unit)
LIBS	Laser-induced breakdown spectroscopy
LIF	Laser-induced fluorescence
LII	Laser-induced incandescence
Li	Lithium
MEDB	Multi—element diffusion burner
mg	Milligram (mass unit)
ml	Milliliters (volume unit)
Mn_x_O_y_	Manganese oxide
ms	Miliseconds (time unit)
MS	Mass spectrometry
mW	Miliwatts (power unit)
N	Newton—force unit
nm	Nanometers (length unit)
NCM	Nickel-rich cathode material
NP	Nanoparticle
N_2_	Nitrogen (molecule)
OV	Oxygen vacancies
Pa	Pascals (pressure unit)
PAH	Polycyclic aromatic hydrocarbon
pH	Potential of hydrogen
PL	Photoluminescence
PS-LIBS	Phase-selective laser-induced breakdown spectroscopy
Pt	Platinum
SAED	Selected area electron diffraction
SAXS	Small-angle X-ray scattering
SE	Secondary electrons
SEM	Scanning electron microscopy
SiO_2_	Silicon oxide
SMPS	Scanning mobility particle size
SSA	Specific surface area
STP	Standard temperature and pressure
TEM	Transmission electron microscopy
TGA	Thermogravimetric analysis
TiO_2_	Titanium oxide
TiO_2_-II	Srilankite phase of TiO_2_
TiRe	Time-resolved
TMS	Tetramethylsilane
TTIP	Titanium isopropoxide
UHV	Ultra-high vacuum
UV-vis-NIR	Ultraviolet-visible-near infrared
VSM	Vibrating sample magnetometer
WO_3_	Tungsten oxide
XPS	X-ray photoelectron spectroscopy
XRD	X-ray diffraction
YSZ	Zirconium acetate + Yttrium acetate
ZnO	Zinc oxide
Zr_x_O_y_	Zirconium oxide
Zst	Stoichiometric mixture fraction
φ	Equivalence ratio
